# Decellularized Periosteum-Derived Hydrogels Promote the Proliferation, Migration and Osteogenic Differentiation of Human Umbilical Cord Mesenchymal Stem Cells

**DOI:** 10.3390/gels8050294

**Published:** 2022-05-10

**Authors:** Shuyi Li, Rongli Deng, Tim Forouzanfar, Gang Wu, Daping Quan, Miao Zhou

**Affiliations:** 1 Department of Stomatology, Guangdong Provincial People’s Hospital, Guangdong Academy of Medical Science, Guangzhou 510080, China; s.li@acta.nl; 2 Department of Oral and Maxillofacial Surgery/Pathology, Amsterdam UMC and Academic Center for Dentistry Amsterdam (ACTA), Amsterdam Movement Science, Vrije Universiteit Amsterdam, 1081 LA Amsterdam, The Netherlands; t.forouzanfar@amsterdamumc.nl; 3 PCFM Laboratory, School of Chemistry and School of Materials Science and Engineering, Sun Yat-sen University, Guangzhou 510006, China; dengrli@mail2.sysu.edu.cn; 4 Department of Oral Cell Biology, Academic Center for Dentistry Amsterdam (ACTA), University of Amsterdam and Vrije Universiteit Amsterdam, 1081 LA Amsterdam, The Netherlands

**Keywords:** periosteum, decellularized extracellular matrix-derived hydrogels, Matrigel, hUCMSCs, osteogenic differentiation

## Abstract

Human umbilical cord mesenchymal stem cells (hUCMSCs) are promising for bone tissue engineering, which have a non-invasive harvesting process, high cell yield, favorable proliferation capacity, and low immunogenicity. However, the osteogenic efficacy of hUCMSCs is relatively lower than that of bone marrow mesenchymal stem cells (BMSCs). Hydrogels from decellularized extracellular matrix (dECM) preserve the biological compositions and functions of natural ECM, which can provide tissue-specific cues to regulate phenotypic expression and cell fate. It is unknown, however, whether hydrogels from periosteum can serve as pro-osteogenic carriers of hUCMSCs. Herein, a decellularized periosteum-derived hydrogel (dPH) was fabricated to reveal the effects of periosteum-specific cues on the bioactivities of hUCMSCs. A widely used non-bone/periosteum-derived ECM hydrogel product, Matrigel, was used as the control group. After decellularization, the absence of nuclei in the histological analysis indicated a successful removal of cellular components, which was also confirmed by DNA content quantification. The storage modulus of dPH increased (from 164.49 ± 29.92 Pa to 855.20 ± 20.67 Pa) with increasing concentration (from 0.5% to 1%). With a highly porous, fibrous microstructure, dPH had a more hydrophilic surface than Matrigel, of which the water contact angle reduced 62.62 ± 0.04%. Furthermore, dPH prominently promoted the initial cellular spreading with a significantly higher cell surface area (1.47-fold), cell spreading length (1.45-fold) and proliferation (approximately 1.05–1.13-fold) of hUCMSCs than those of Matrigel. Additionally, dPH was conducive to cell migration, whereas no cells migrated to Matrigel in the Transwell model. Compared with those of the Matrigel group, the osteogenesis-related genes expression levels (runt-related transcription factor 2 (RUNX2), alkaline phosphatase (ALP), osteopontin (OPN), and osteocalcin (OCN)) and mineralized matrix formation (9.74-fold) of the hUCMSCs significantly increased in the dPH group. Our study indicated that dPH could provide a pro-osteogenic microenvironment for hUCMSCs, thereby revealing a promising application potential to repair bone defects.

## 1. Introduction

Mesenchymal stem cell (MSC)-based constructs have been considered promising alternatives to autologous bone grafts in repairing bone defects [1,2]. MSCs are multipotent stem cells and can differentiate into diverse cell types, such as osteoblasts, chondrocytes, and adipocytes under specific stimuli from culture media or biomaterials [3,4]. MSCs can be isolated from various tissues, such as bone marrow, umbilical cord, and adipose tissue. Human bone marrow mesenchymal stem cells (hBMSCs) are the most widely used stem cells to promote bone regeneration [5]. However, the invasive harvesting process, low yield, and slow proliferation rate of hBMSCs restrict their clinical applications [6,7]. In contrast, human umbilical cord mesenchymal stem cells (hUCMSCs) are primitive and possess a non-invasive harvesting procedure, an abundant source, and a high cell yield [8], which secure a sufficient number of cells for bone tissue engineering (BTE). HUCMSCs also have a significantly higher proliferation capacity than hBMSCs and maintain high activity after multiple passages [9,10]. Moreover, hUCMSCs show lower expression levels of human leukocyte antigen (HLA) I/II and a higher production of tolerogenic interleukin-10 (IL-10), transforming growth factor-β (TGF-β), and HLA-G than hBMSCs. These indicate low immunogenicity and a strong immunosuppressive capacity of hUCMSCs and thereby support the application of allogenic hUCMSCs [11,12]. All these characteristics make hUCMSCs promising MSCs for BTE. However, a series of in-vitro studies have shown that hUCMSCs form a less mineralized extracellular matrix, the final osteogenic differentiation marker, than that of hBMSCs in osteogenic differentiation medium [10,13,14], which suggests a relatively lower osteogenic capacity of hUCMSCs. Sudo et al. show that almost no mineralized extracellular matrix is formed in hUCMSCs when being cultured on collagen type I-coated plastic dishes in osteogenic medium (OM) for 28 days [15]. Ciavarella et al. find mineralized extracellular matrix is detected in osteogenically-committed hUCMSCs after 40 days [16]. The microenvironment where hUCMSCs reside serves as an important regulatory role in the process of proliferation and osteogenic differentiation [17]. When OM contains osteoinductive growth factors, such as bone morphogenetic protein-2 (BMP2) and BMP7, the osteogenic property of hUCMSCs is significantly improved, and the expression levels of osteogenic genes are comparable to those of hBMSCs [17,18]. Our group adopted a biomimetic strategy of co-culturing osteogenically- and angiogenically-committed hUCMSCs in certain ratios and screened culture media to enhance their osteogenic efficacy [19]. These studies indicate that a strong osteogenic microenvironment is conducive to the osteogenic differentiation of hUCMSCs.

Natural extracellular matrix (ECM) has garnered extensive attention because it provides essential physical support for cells and initiates crucial biological signals which are necessary for tissue morphogenesis, differentiation, and homeostasis [20]. ECM is mainly composed of fibrous proteins (such as collagen type I, II, III, IV, VI, X, and elastin) and glycoproteins (such as proteoglycans, fibronectin, and laminin) [21]. It possesses abundant cell-recognition sites and various protein-adhesive domains to localize and deliver growth factors (such as BMP2, vascular endothelial growth factor (VEGF), and TGF-β), which can regulate cellular activities and phenotypic expression [21,22]. Given their structural similarity to natural ECM, tunable physicochemical properties, and high operability, hydrogels that are derived from ECM components, such as collagen and complex mixtures of ECM proteins (such as Matrigel), are widely used in tissue engineering and regenerative medicine [23,24]. Matrigel is derived from the basement membrane of murine Engelbreth-Holm-Swarm (EHS) tumor and contains a mixture of ECM proteins, such as laminin, collagen type IV, perlecan, entactin, and growth factors [25]. It has been extensively applied to culture cells in 2D/3D ways and promotes cell growth and differentiation [26]. As a non-bone-specific hydrogel, Matrigel primarily functions to provide a 2D/3D culture platform for adipose tissue-derived MSCs. Matrigel-coated culture plates show significantly enhanced extracellular matrix mineralization compared to that of polystyrene culture plates [27]. However, due to its origin from mouse tumors, the safety of Matrigel is a major concern. Furthermore, Matrigel may not contain all the necessary biological cues for osteogenesis. Therefore, an ECM-derived hydrogel specific for osteogenesis with a safe origin is in high demand, which can not only deliver cells to defect areas, but also provide a pro-osteogenic microenvironment to support and regulate cellular activities.

ECM hydrogels derived from decellularized tissues have been widely applied to repair tissue injuries or defects, such as intervertebral discs [28], peripheral nerves [29], and bones [30]. It has been established that the ECM hydrogel bears a tissue-specific induction property. For example, ECM hydrogels from decellularized nucleus pulposus (NP) induce hBMSCs to differentiate into NP-like cells, while the hydrogels from decellularized annulus fibrosus (AF) induce the formation of AF-like cells [28]. Similarly, ECM hydrogels from peripheral nerve matrix are more effective in supporting myelination, whereas hydrogels from spinal cord promote synapse formation [31]. As a connective tissue membrane covering the outer surface of bone, periosteum is essential to regulate bone development and regeneration [32]. Periosteum contains bone-forming related cells (such as osteoprogenitor cells and osteoblasts), growth factors (such as BMPs and VEGF), and a specific 3D ECM microenvironment [33]. It can facilitate bone regeneration in acute bone fractures and critical-sized bone defects [34,35]. ECM from decellularized periosteum preserves the functional components (such as collagen and glycosaminoglycans (GAGs)) and has superior osteogenic activities both in vitro and in vivo [36]. Therefore, ECM hydrogels from decellularized periosteum resemble the functions of periosteum, which significantly promote the osteogenic differentiation of mouse BMSCs compared with pure collagen hydrogels [30]. Whether ECM hydrogels from periosteum can serve as a pro-osteogenic carrier for hUCMSCs is unknown.

We hypothesized that the decellularized periosteum-derived hydrogel (dPH) could be used as a favorable carrier of hUCMSCs and provide a periosteum-specific pro-osteogenic microenvironment to promote their osteogenic differentiation. In this study, dPH was made from decellularized periosteum (DP). Physicochemical properties, such as the microstructure, gelation kinetics, rheological property, and hydrophilicity were evaluated. Furthermore, hUCMSCs were seeded onto dPH and Matrigel-coated coverslips to assess cellular activities and osteogenic differentiation. These studies would provide fundamental results for the application of dPH-carried hUCMSCs to promote the repair of bone defects.

## 2. Results

### 2.1. Characterization of DP

**Figure 1:**
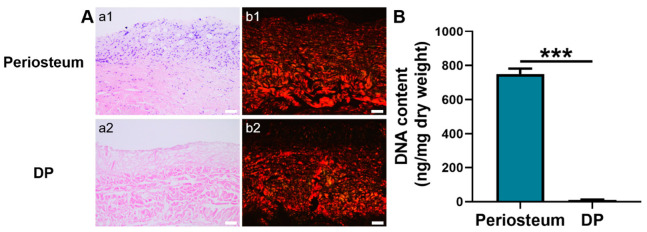
Characterization of periosteum and DP. (**A**) Histological analysis. a1–a2 H&E staining. b1–b2 Picrosirius red staining. (**B**) DNA content quantification. Scale bar = 50 μm. *** *p* < 0.001.

Hematoxylin and eosin (H&E) staining showed that the native periosteum had a bilayer ECM structure with dark-blue stained nuclei. In contrast, the ECM structure of DP was loosely disposed and no dark-blue stained nuclei were detected (Figure 1A), suggesting an efficient removal of cellular components. Besides, DNA quantitative analysis showed that the DP had a significantly lower DNA content (10.75 ± 1.4 ng/mg) than native periosteum (749.67 ± 32.50 ng/mg) (*p* < 0.001). Furthermore, the DNA content in the DP was lower than the internationally required criterion of 50 ng/mg (Figure 1B) [37]. Under a polarized microscope, the picrosirius red-stained native periosteum displayed closely packed, red-stained collagen type I and green-stained collagen type III fibers [38]. A similar phenomenon was observed in the DP although its fibrous layer was not intensely stained with red dye.

### 2.2. Turbidimetric Gelation Kinetics

**Figure 2:**
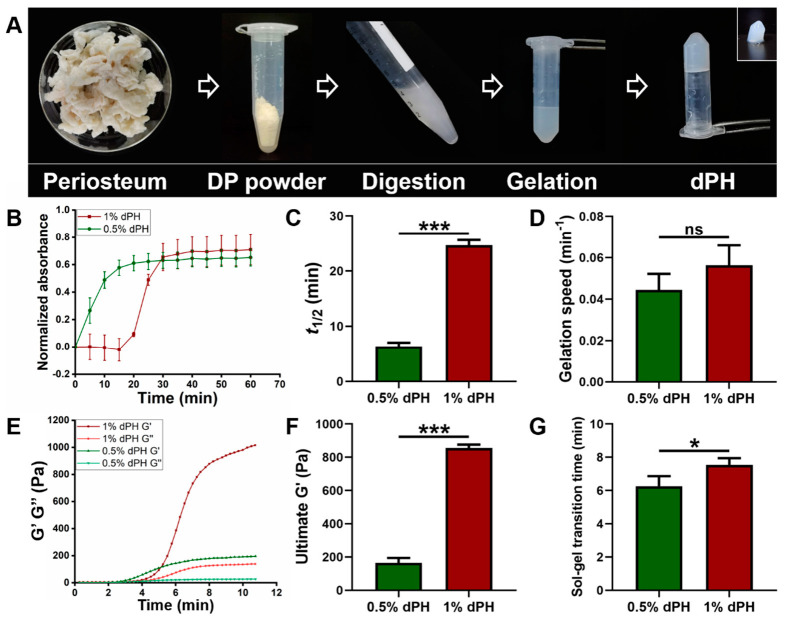
The fabrication process of dPH, its turbidimetric gelation kinetics, and rheological property. (**A**) Fabrication process of dPH. (**B**) Representative normalized absorbance curve of 0.5% and 1% dPH. (**C**) Time to reach 50% complete gelation of 0.5% and 1% dPH. (**D**) Gelation speed of 0.5% and 1% dPH. (**E**) Representative rheological property curve of dPH. G′ represents the storage modulus, and G″ refers to the loss modulus. (**F**) Ultimate storage modulus of 0.5% and 1% dPH. (**G**) Sol-gel transition time of 0.5% and 1% dPH. ns, no significant difference, * *p* < 0.05, *** *p* < 0.001.

As illustrated in Figure 2A, the DP was milled into powder and underwent the digestion process. After gelation, dPH was semi-transparent and self-supportive (inset image) (Figure 2A). The turbidimetric gelation kinetics of 1% dPH was a sigmoidal shape whereas the curve of 0.5% dPH was an exponential shape (Figure 2B). Moreover, 1% dPH had an average lag phase of 18.32 ± 0.40 min. *T*_1/2_ of 0.5% dPH and 1% dPH were 6.35 ± 0.70 min and 24.66 ± 0.99 min, respectively (*p* < 0.001) (Figure 2C). Additionally, 0.5% dPH shared a similar gelation speed with 1% dPH (0.044 ± 0.007/min and 0.056 ± 0.01/min, respectively) (Figure 2D). The maximum turbidity value of 1% dPH was higher than that of 0.5% dPH.

### 2.3. Rheological Property of dPH

Different rheological properties of 1% and 0.5% dPH can be observed in Figure 2E–G. G′ and G″ of both 1% and 0.5% dPH increased as the temperature rose to 40 °C. The 1% dPH had a longer lag period than did 0.5% dPH. The sol-gel transition period of 1% dPH lasted 7.55 ± 0.40 min, and the G′ was 855.20 ± 20.67 Pa. In comparison, the sol-gel transition time of 0.5% dPH was 6.26 ± 0.60 min (*p* < 0.05) (Figure 2F,G), and the G′ was 164.49 ± 29.92 Pa (*p* < 0.001). In each concentration of dPH, G′ was higher than G″.

### 2.4. Characterization of dPH and Matrigel

**Figure 3:**
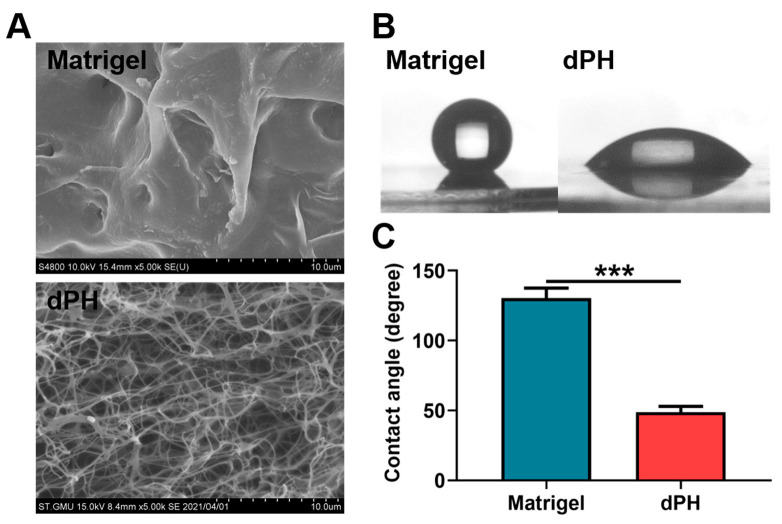
Morphological observations and hydrophilic study of Matrigel and dPH. (**A**) Representative images of Matrigel and dPH observed using SEM. (**B**) Representative optical images of hydrophilic study. (**C**) Quantitative analysis of WCA. *** *p* < 0.001.

Scanning electron microscopy (SEM) revealed that Matrigel had a dense microstructure while dPH was porous with an interwoven network. DPH had an average pore size of 2.07 ± 0.63 μm (Figure 3A). In the hydrophilic study, Matrigel had a hydrophobic surface with a water contact angle (WCA) of 130.45 ± 7.17°. In contrast, dPH was hydrophilic with a WCA of 48.63 ± 4.26°, which was significantly lower than that of Matrigel (*p* < 0.001) (Figure 3B,C).

### 2.5. Cellular Activities

**Figure 4:**
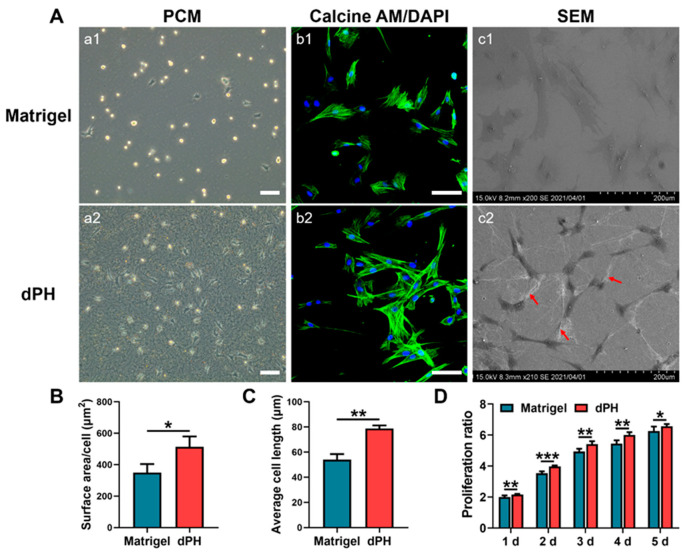
Morphological analysis and proliferation rate of hUCMSCs cultured on Matrigel and dPH. (**A**) Representative images of hUCMSCs cultured on Matrigel and dPH. (a1,a2) Phase contrast micrographs of hUCMSCs cultured on Matrigel and dPH for 24 h. (b1,b2) Cytoskeletal staining of hUCMSCs cultured on Matrigel and dPH for 48 h. (c1,c2) SEM images of hUCMSCs cultured on Matrigel and dPH for 48 h. The red arrows indicate nanofibers in dPH. (**B**,**C**) Cell surface area and length of hUCMSCs when being cultured on Matrigel and dPH for 24 h. (**D**) The proliferation rate of hUCMSCs when cultured on Matrigel and dPH. Scale bar = 100 μm. * *p* < 0.05, ** *p* < 0.01, *** *p* < 0.001.

Initial cellular activities were recorded by observing the morphologies of hUCMSCs on the two hydrogels. After seeding the cells for 24 h, most of the hUCMSCs on Matrigel had not spread and displayed a round appearance as shown in a phase-contrast microscopy (PCM) (Figure 4A). In contrast, hUCMSCs on dPH had pseudopodia and interacted with surrounding dPH fibers. HUCMSCs on dPH possessed a significantly higher cell spreading surface area and cell length than those on Matrigel (*p* < 0.05) (Figure 4B,C). The morphologies of hUCMSCs were observed 48 h later using confocal laser scanning microscopy (CLSM) and SEM. Immunofluorescent staining showed that the cells on dPH stretched to form a spindle-shaped structure. SEM displayed that hUCMSCs surrounded by dPH fibers elongated and became slender. In contrast, the cells on Matrigel began to spread into a polygonal shape. Some of the cells had pseudopodia and attached to the surrounding Matrigel (Figure 4(Ac1)). In the cell proliferation test, hUCMSCs grew more rapidly on dPH than those on Matrigel at all observed time points (*p* < 0.05) (Figure 4D).

### 2.6. Transwell Chemotaxis Assay

**Figure 5:**
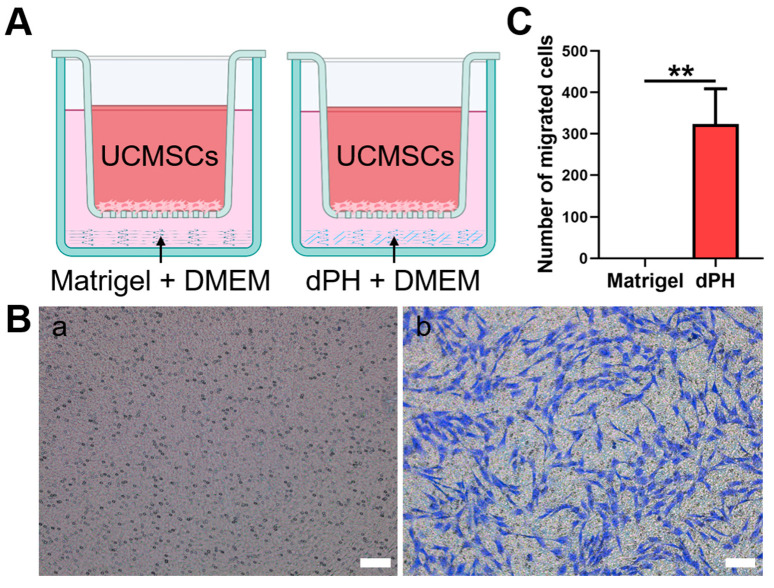
The chemotactic assay of Matrigel and dPH. (**A**) A schematic illustration of the assay. (**B**) HUCMSCs migrated through the upper chamber membranes of Transwell to Matrigel (a) and dPH (b) after 12 h. (**C**) Number of migrated cells in dPH and Matrigel. Scare bar = 100 μm. ** *p* < 0.01.

The chemotaxis of hUCMSCs was evaluated, which were not in direct contract with dPH or Matrigel (Figure 5). In the Matrigel-coated wells, no hUCMSCs migrated to the lower chamber. In contrast, a significantly higher number of migrated cells were observed in dPH group (*p* < 0.01), indicating that dPH exhibited a strong chemotactic effect.

### 2.7. Osteogenic Activities

**Figure 6:**
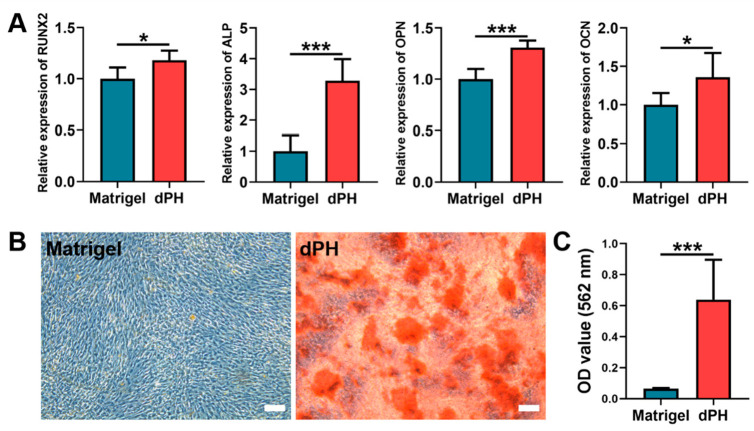
In-vitro osteogenic differentiation of hUCMSCs after being cultured on Matrigel and dPH for 14 days. (**A**) Expression levels of osteogenesis-related genes (RUNX2, ALP, OPN, and OCN). (**B**) ARS staining of the mineralized matrix formation of hUCMSCs cultured on Matrigel and dPH. (**C**) Quantitative analysis of ARS. Scale bar = 100 μm. * *p* < 0.05, *** *p* < 0.001.

On day 14, qPCR results showed that the hUCMSCs cultured on dPH had significantly higher expression levels of runt-related transcription factor 2 (RUNX2), alkaline phosphatase (ALP), osteopontin (OPN), and osteocalcin (OCN) than those on Matrigel (Figure 6A). As to the alizarin red S staining (ARS), a consistently higher mineralized matrix (9.74-fold) was observed in the dPH group than that in the Matrigel group, which was also confirmed by the quantitative analysis (Figure 6B,C). The aforementioned results indicated that hUCMSCs cultured on dPH were more inclined to osteogenic differentiation.

## 3. Discussion

The major concern of MSC-based therapy in BTE is the survival and bioactivity of seed cells. A tissue-specific ECM microenvironment is of paramount importance to regulate cellular activities. We fabricated a novel decellularized periosteum-derived hydrogel (dPH), aiming to provide a pro-osteogenic microenvironment for hUCMSCs to improve the osteogenic efficacy. DPH had a fibrous microstructure with a significantly higher hydrophilic surface than that of the basement membrane-derived Matrigel. Furthermore, dPH exhibited a prominent effect on cellular spreading, migration, and proliferation. Moreover, enhanced osteogenesis-related genes expression levels and mineralized matrix formation were observed in hUCMSCs when being cultured on dPH. These results showed that dPH could provide a favorable osteogenic microenvironment for hUCMSCs, indicating a promising application potential for the repair of bone defects.

The microenvironment surrounding cells provides both biophysical and biochemical signals which will affect cellular bioactivities. Viscoelastic properties (such as gelation kinetics and gel stiffness) are crucial to the application of ECM hydrogels [23]. Turbidimetric gelation kinetics indicate the process of hydrogel formation: collagen monomeric components self-assemble into fibrils and then into collagen fibers, which will further interweave with themselves and other ECM molecules, thus forming a fibrous network [39]. This self-assembly process of collagen type I can be altered by collagen type V, GAGs, and proteoglycans (PGs) [40,41]. Decellularized brain-derived ECM hydrogels have an exponential shape of gelation kinetics with an approximated 3-min lag in fibrillogenesis [39]. The gelation kinetics curve is consistent with that of the 0.5% dPH. However, no lag phase was observed in 0.5% dPH, which might be attributed to a longer time interval between tests. The gelation kinetics curves of most other ECM-derived hydrogels present a sigmoid shape. In these hydrogels, decellularized cartilage-derived ECM hydrogels have a short lag phase of approximately 8 min, while the lag phases of hydrogels from the dermis and urinary bladder range from 15 to 25 min, which is consistent with that of 1% dPH [42,43,44]. Rheology is extensively used to determine the stiffness and gelation time of hydrogels [23]. G′ represents the stiffness, and G″ refers to the viscosity of a specific hydrogel [45]. After complete gelation, G′ was higher than G″, indicating that dPH formed a solid structure. The stiffness increased from 164.49 ± 29.92 Pa to 855.20 ± 20.67 Pa following a rising concentration in dPH. The values of mechanical property were lower than that of the range in which osteogenesis usually occurs (G′ of 3.7–10.7 kPa) [46]. However, gelatin-based hydrogels with a lower stiffness (G′ of 538 ± 91 Pa) support better osteogenesis with a more intense mineralized matrix formation than stiff hydrogels (G′ of 7263 ± 287 Pa) [46]. The gelation kinetics and G′ of dPH could be adjusted by its concentration, which indicated that the lag time and mechanical stiffness of dPH might be tailored for specific applications in repairing bone defects.

Apart from the mechanical property, the biochemical characteristics also support the bioactivities of dPH. Picrosirius red staining qualitatively showed collagen was well-preserved in the decellularized periosteum. The effective preservation of fibronectin and GAGs in the decellularized periosteum were also observed in our previous study [47]. Preserved collagen and fibronectin can provide binding sites to facilitate cell adhesion, spreading, migration, and proliferation [48,49]. When dPH and Matrigel were co-cultured with hUCMSCs, a significantly higher initial cell spreading surface area (1.47-fold), cell spreading length (1.45-fold), and growth (1.05–1.13-fold) were observed in dPH than in Matrigel. This may be attributed to the following mechanisms: first, we showed that dPH was more hydrophilic than Matrigel, which might have exerted a positive effect on initial cell spreading on dPH; second, periosteal ECM hydrogels contain various cell adhesion-related proteins, such as fibronectin, fibrillin-1, vitronectin, and thrombospondin 4 [30], while Matrigel lacks these proteins [25,50]. Some other studies have also reported a higher cell proliferation in ECM hydrogels than in Matrigel. For example, Miao et al. show that the proliferation of chondrocytes is significantly higher in collagen than in Matrigel [51]. Similarly, a decellularized brain matrix markedly supports and promotes dendritic formation of neurons as opposed to Matrigel [52].

As a promising alternative cell source to BMSCs in BTE, hUCMSCs have been studied in different scenarios with conflicting osteogenic outcomes. Chen et al. find that hUCMSC-based macro-porous calcium phosphate cement (CPC) constructs have similar bone regeneration properties to those of hBMSC-based CPC constructs in vivo [53]. Kouroupis et al. directly compare the osteogenic properties of hBMSCs and hUCMSCs and find that the mineralized matrix formation is significantly lower in hUCMSCs than that in hBMSCs [13], indicating a relatively lower osteogenic ability of hUCMSCs. HUCMSCs tend to express more angiogenesis- and growth-related genes, while hBMSCs express more osteogenic genes. Furthermore, hUCMSCs are more primitive than BMSCs, and are not so sensitive to environmental stimulations [54]. Therefore, when hUCMSCs were cultured on Matrigel in OM, no mineralized nodules formed after 14 days. On the contrary, distinctly elevated mineralized matrix formation (9.74-fold) and gene expression levels were found when hUCMSCs were cultured with dPH, which might be due to the soluble bioactive agents in dPH. For example, periostin can significantly promote the migration and mineralized matrix formation of MSCs [55]. ECM hydrogels also serves as pools of various growth factors, such as VEGF and TGF-β [56,57]. Whether dPH contains all of these bioactive factors will be analyzed in our future study.

Recruiting cells from local sites is one of the main methods to gather sufficient cells to promote tissue repair. Fibronectin, vitronectin, and collagen type I can induce the dose-dependent chemotaxis of hMSCs, and fibronectin has the strongest chemotactic response [58]. ECM scaffolds derived from small intestinal submucosa are able to recruit cells to defect sites [59]. Besides, the degradation products of ECM can regulate the migration of progenitor cells [60]. Compared with Matrigel, dPH induced a significantly higher migration of hUCMSCs in the Transwell model. This indicated that there were soluble ECM proteins in dPH that might guide cells towards defects when being implanted in vivo. The secreted protein acidic and rich in cysteine (SPARC) and insulin-like growth factor-binding 5 in periosteal ECM hydrogels have shown positive effects on cell migration [30]. To date, it remains unclear how the ECM regulates cellular activities. Our future studies will therefore focus on the extraction of dPH proteins and the characterization of their functions and related mechanisms. 

## 4. Conclusions

The decellularized periosteum-derived hydrogel had a fibrous morphology and hydrophilic surface. It provided both unique biophysical and biochemical signals to regulate the bioactivities of hUCMSCs. Furthermore, dPH exhibited a prominent effect on promoting the initial cellular spreading, migration, and proliferation of hUCMSCs. With an improved simulation of the periosteal microenvironment, dPH was associated with enhanced osteogenesis-related genes expression and mineralized matrix formation of hUCMSCs than those of Matrigel. All these results suggested that dPH could be used as a favorable carrier for hUCMSCs to promote bone regeneration.

## 5. Materials and Methods

### 5.1. Fabrication of the Decellularized Periosteum-Derived Hydrogel (dPH)

Periostea were harvested from fresh porcine femoral bones in a slaughterhouse. Under sterilized conditions, the periosteal tissues were washed with deionized water and subjected to freeze-thaw cycles (−80 °C–37 °C) for three times. Thereafter, the periosteal tissues were decellularized by 1% Triton X-100, 1% sodium dodecyl sulfate (Sigma-Aldrich Corp., St. Louis, MO, USA), and 50 U/mL DNase (Sigma-Aldrich Corp., St. Louis, MO, USA) successively. The resultant tissues were washed thoroughly and sterilized in 75% medical-grade ethanol before being lyophilized. The lyophilized decellularized tissues were milled into powder and digested in 1 mg/mL pepsin solution (dissolved in 0.01 M HCl, Sigma-Aldrich Corp., St. Louis, MO, USA) for 7 h under constant stirring. The pre-gel solution was centrifuged (3000 rpm, 4 °C for 10 min) to precipitate and remove undigested particulate residue. To construct dPH, the fresh pre-gel solution would be neutralized using precooled 1M NaOH and HCl solutions to a pH of 7.4. Subsequently, 10× phosphate buffer saline (PBS, 1/9 of final volume) was used to equilibrate its salinity. The neutralized dPH solution was incubated at 37 °C for 15–30 min to induce gelation. Commercially available growth factor reduced Matrigel^®^ basement membrane matrix (7.6 mg/mL in protein concentration; 356,231, Corning, NY, USA) was used as the control group to evaluate cellular responses.

### 5.2. Characterization of the DP

ECM structure of DP and the presence of nuclei were assessed by H&E staining, picrosirius red staining, and DNA content quantification. As to H&E staining and picrosirius red staining, native periosteum and DP were fixed in 10% neutral buffered formalin solution, dehydrated, embedded in paraffin, and sectioned into 5-μm slices. The slices were stained by H&E solution and picrosirius red.

The nucleic acid concentration was examined by Quant-iT™ PicoGreen™ dsDNA Reagent (Invitrogen, Waltham, MA, USA). In brief, the lyophilized DP and native periosteum (n = 3) were digested with Proteinase K, purified with the TIANamp Genomic DNA kit (Tiangen, Beijing, China), and then incubated with Picogreen according to the manufacturer’s instructions. Total DNA content was tested by a microplate reader (Synergy™ HTX, Biotek, Winooski, VT, USA) (excitation wavelength: 485 nm; emission wavelength: 528 nm).

### 5.3. Turbidimetric Gelation Kinetics

The turbidimetric gelation kinetics were determined spectrophotometrically as previously described [44,61]. Pre-gel dPH solutions with concentrations of 1% and 0.5% were transferred to a pre-cold 96-well plate at 100 μL per well in triplicates. The plates were placed in an incubator with a constant temperature of 37 °C. The turbidity value of each well was measured at 405 nm every 5 min for 1 h using the microplate reader. The absorbance values were recorded and normalized according to the following equation: normalized absorbance = (A–A_0_)/(A_max_–A_0_), where A is the absorbance at a pre-determined time, A_0_ is the initial absorbance and A_max_ is the maximum absorbance. The lag phase was calculated as the intercept of the linear portion of the curve with an absorbance of 0. *T*_1/2_ referred to the time needed to reach 50% of the maximum absorbance value. The gelation speed represented the slope of the linear portion of the gelation curve.

### 5.4. Rheological Property of dPH

The rheological properties of dPH at different concentrations (1% and 0.5%, n = 3) were assessed with a strain-controlled rheometer (Thermo Scientific, HAAKE MARS III, Karlsruhe, Germany) using an oscillatory time sweep. Briefly, pre-cooled pH and ion-balanced dPH solution (350 μL) was transferred to the plate of the rheometer with a homogeneous distribution. The gap distance was 0.9 mm. The frequency was set to 1 Hz with a strain of 1%. The temperature increased from 20 °C to 40 °C at 210 °C/min. Storage modulus (G′) and loss modulus (G″) were recorded.

### 5.5. Morphologies of dPH and Matrigel

The microstructures of dPH and Matrigel were observed using SEM (S3400N, Hitachi, Tokyo, Japan). Before the test, the two hydrogels were fixed in 2.5% glutaraldehyde for 1 h and subjected to thorough washing in deionized water. The resultant samples were dehydrated in gradient concentrations of ethanol (30%, 50%, 80% and 100% for 15 min each). After that, the samples were immersed in deionized water and freeze-dried. The lyophilized samples were torn to generate a fracture surface, sputter-coated with Au–Pd, and observed under SEM.

### 5.6. Hydrophilic Properties

A water contact angle test was used to measure the hydrophilicity of dPH and Matrigel with a precise goniometer (DSA 100, KRÜSS GmbH Co., Hamburg, Germany). A 3.5 μL drop of water was added on dPH or Matrigel pre-coated coverslips (φ = 14 mm). All the tests were repeated six times.

### 5.7. Evaluations of Cellular Activities

#### 5.7.1. Cell Culture

Commercially available hUCMSCs (OriCell^®^, Cyagen, Guangzhou, China) were used to evaluate the cellular activities on the hydrogels. HUCMSCs were cultured in a specialized hMSC medium (P4–P6, Nuwacell Ltd., Anhui, China) and incubated in a humidified atmosphere with 5% CO_2_ at 37 °C. The medium was changed every 3–4 days. When the cells reached 70–80% confluence, hUCMSCs were detached, counted, and used for subsequent tests.

#### 5.7.2. Cellular Morphologies on the Hydrogels

Before seeding the cells, dPH (7.6 mg/mL) and Matrigel were coated on coverslips (φ = 14 mm; 150 μL, n = 3) and incubated at 37 °C for 30 min to allow thorough gelation. HUCMSCs (2 × 10^4^/mL) were seeded onto the coated coverslips and cultured in the hMSC medium. The cellular morphologies were recorded 24 h later using PCM (Leica, Wetzlar, Germany) with three different regions of interest. The cell spreading surface area and cell length were measured using ImageJ 1.46r software (National Institutes of Health, Bethesda, MD, USA). The cells were fixed in 4% paraformaldehyde solution 48 h later and observed using SEM and CLSM (Leica, Wetzlar, Germany). Before being observed under SEM, the cells were dehydrated in gradient concentrations of ethanol and sputter-coated with Au–Pd. As to immunofluorescent staining of the cytoskeleton, the cells were permeabilized with 0.1% Triton X-100 solution for 5 min. After thorough washing, rhodamine phalloidin-labeled FITC solution (Cytoskeleton, Denver, CO, USA) was used to visualize F-actin in the cells (30 min), and DAPI solution was applied to stain the nuclei (5 min).

#### 5.7.3. Cell Proliferation

For the cell proliferation analysis, 100 μL hUCMSCs (5 × 10^4^/mL) were seeded onto dPH (7.6 mg/mL) and Matrigel-coated 96-well plate (15 μL, n = 6). They were cultured in hMSC medium continuously for 5 days. The Alamarblue assay was used to assess metabolic activity. Each day before the assay, 100 μL of new culture medium with 10 μL Alamarblue reagent (Invitrogen™, Waltham, MA, USA) was added to each well. After the samples had been incubated at 37 °C for 3 h, the optical density (OD) values were recorded at 570 nm and 600 nm using the microplate reader. The rate of cell proliferation was calculated according to the manufacturer’s protocol.

#### 5.7.4. Transwell Chemotaxis Assay

To assess the migration of hUCMSCs to dPH and Matrigel, a Transwell-24 plate with a pore size of 8 μm (Costar, Corning, NY, USA) was used. DPH and Matrigel (80 μL) were pre-coated in the lower chambers (n = 4). Thereafter, 100 μL of 2 × 10^5^/mL hUCMSCs were seeded into the upper chambers and cultured in high glucose Dulbecco’s modified Eagle’s medium (DMEM) containing 1% fetal bovine serum (FBS) and 1% penicillin-streptomycin (PS). 600 μL of standard DMEM solution was added to the lower chamber. After incubation for 12 h, the non-migrating cells in the upper chamber were scraped away. The migrated cells at the bottom of the upper chamber membrane were then fixed in 4% paraformaldehyde solution. 0.1% crystal violet (Beyotime Biotechnology, Shanghai, China) was applied to stain the cells for 20 min. The migrated cells were captured and counted.

#### 5.7.5. Osteogenic Activity

To evaluate the osteogenic responses of hUCMSCs on dPH (7.6 mg/mL) and Matrigel, hUCMSCs (2 × 10^5^/well) were seeded onto dPH and Matrigel coated 6-well plates. They were cultured in the osteogenic medium, which contained DMEM, 10% FBS, 1% PS, 100 nmol/L dexamethasone, 10 mmol/L β-glycerophosphate, and 50 μg/mL L-ascorbic acid. The medium was changed every 3 days.

After being cultured in the osteogenic medium for 14 days, hUCMSCs were harvested to test the genes expression profiles by quantitative reverse transcription-polymerase chain reaction (qRT-PCR) assay (n = 3). Briefly, TRIzol^®^ (Invitrogen, Waltham, MA, USA) was used to extract total RNA. The quality and concentration of isolated RNA were evaluated using NanoDrop 2000 spectrophotometer (Thermo Fisher Scientific, Waltham, MA, USA) at 260/280 nm. Subsequently, RNA was reverse-transcribed into cDNA via PrimeScript^TM^ RT Master Mix (TaKaRa, Beijing, China). QPCR analysis was conducted using TB Green^TM^ Premix Ex Taq^TM^ II (TaKaRa, Beijing, China) and synthesized primers (Generay, Shanghai, China) in a Real-Time fluorescent qPCR system (Bio-Rad, Hercules, CA, USA). The expression levels of RUNX2, ALP, OPN, and OCN were normalized to housekeeping gene glyceraldehyde 3-phosphate dehydrogenase (GAPDH). The primer sequences are listed in Table 1.

ARS was used to evaluate osteogenic mineralization (n = 6). After thorough washing, hUCMSCs were fixed in 4% paraformaldehyde solution for 30 min. ARS reagent (0.2%, pH 8.3) was added and cocultured for 10 min. Non-specific staining was removed by repeated washing. The mineralization nodules were recorded using the phase-contrast microscopy. For quantitative evaluation, 10% hexadecyl pyridinium chloride monohydrate (CPC) was added to dissolve the mineralized nodules. The colorimetric absorbance was measured at 562 nm using the microplate reader.

### 5.8. Statistical Analysis

Data were expressed as the mean ± standard deviation (SD). Statistical analysis was performed using SPSS 20.0 software (SPSS, Chicago, IL, USA). The difference between the two studied groups was assessed by Student’s t-test. *p* < 0.05 was considered to indicate statistical significance.

## Figures and Tables

**Table 1:** The sequences of primers used for qRT-PCR analysis.

No.	Primer Name	Sequences
1	RUNX2	sense: TACTATGGCACTTCGTCAGGA antisense: GATTCATCCATTCTGCCACTA
2	ALP	sense: GGCTGTAAGGACATCGCCTA antisense: GGGTCAAGGGTCAGGAGTTC
3	OPN	sense: GCTAAACCCTGACCCATC antisense: CTTTCGTTGGACTTACTTGG
4	OCN	sense: AGGGCAGCGAGGTAGTGAAG antisense: CTCCTGAAAGCCGATGTGGT
5	GAPDH	sense: GCACCGTCAAGGCTGAGAAC antisense: TGGTGAAGACGCCAGTGGA

## Data Availability

Data will be available upon request from the corresponding author.
